# Seeding of epithelial cells into circulation during surgery for breast cancer: the fate of malignant and benign mobilized cells

**DOI:** 10.1186/1477-7819-4-67

**Published:** 2006-09-26

**Authors:** Oumar Camara, Andreas Kavallaris, Helmut Nöschel, Matthias Rengsberger, Cornelia Jörke, Katharina Pachmann

**Affiliations:** 1 Frauenklinik der Friedrich Schiller Universtiät Jena, Bachstrasse 18, D-07740 Jena, Germany; 2 Klinik für Innere Medizin II der Friedrich Schiller-Universität Jena, Erlanger Allee 101 D-07747 Jena, Germany; 3 Transfusionsmedizinisches Zentrum Bayreuth Kurpromenade 2, D-95448 Bayreuth, Germany

## Abstract

**Background:**

Surgery of malignant tumors has long been suspected to be the reason for enhancement of growth of metastases with fatal outcome. This often prevented surgeons from touching the tumor if not absolutely necessary. We have shown in lung cancer patients that surgery, itself, leads to mobilization of tumor cells into peripheral blood. Some of the mobilized cells finding an appropriate niche might grow to form early metastases. Monitoring of tumor cell release during and the fate of such cells after surgery for breast cancer may help to reveal how metastases develop after surgery.

**Method:**

We used the MAINTRAC^® ^analysis, a new tool for online observation of circulating epithelial cells, to monitor the number of epithelial cells before, 30 min, 60 min, three and seven days after surgery and during subsequent variable follow up in breast cancer patients.

**Results:**

Circulating epithelial cells were already present before surgery in all patients. During the first 30–60 min after surgery values did not change immediately. They started increasing during the following 3 to 4 days up to thousand fold in 85% of treated patients in spite of complete resection of the tumor with tumor free margins in all patients. There was a subsequent re-decrease, with cell numbers remaining above pre-surgery values in 58% of cases until onset of chemotherapy. In a few cases, where no further therapy or only hormone treatment was given due to low risk stage, cell numbers were monitored for up to three years. They remained elevated with no or a slow decrease over time. This was in contrast to the observation in a patient where surgery was performed for benign condition. She was monitored before surgery with no cells detectable. Epithelial cells increased up to more than 50 000 after surgery but followed by a complete reduction to below the threshold of detection.

**Conclusion:**

Frequently before but regularly during surgery of breast cancer, epithelial cells are mobilized into circulation. Part of these cells, most probably normal or apoptotic cells, are cleared from the circulation as also shown to occur in benign conditions. After resection even if complete and of small tumors, cells can remain in the circulation over long times. Such cells may remain "dormant" but might settle and grow into metastases, if they find appropriate conditions, even after years.

## Background

Surgery of malignant breast tumors aims at eliminating most of the malignant cells. Adjuvant therapy is applied subsequently in most cases due to the assumption that not all malignant cells have been removed. This approach has proven successful also after 30 years of survey [1]. The question arises when these cells have been mobilized into circulation. Klein *et al *[2] claim that the low genetic variation of the circulating cells as compared to the tumor itself indicates that cells have left the tumor at an early stage. We can, indeed, detect circulating tumor cells already before surgery treatment in all patients so far assessed [3]. "Dormant" tumor cells can be present in the circulation for years without leading to relapse [4,5]. In a report from the data from 30 years of survey [6] it is assumed that early relapses in a subpopulation of breast cancer patients are due to preexisting dormant metastases which can be activated and are induced to growth and/or vascularization by the act of surgery. Recent calculations of the hazard of recurrence in a retrospective study are indicative of acceleration of the metastatic process in some patients by the surgical resection of the primary breast cancer. It is assumed that this phenomenon may be due to release of preexistent tumor cells or of micrometastatic single cells and avascular foci present in the patients before diagnosis from dormancy. The detection of such "dormant" cells in bone marrow seems to have impact on prognosis and relapse free survival [7]. They can be detected in 25% of patients, 40% of which will relapse whereas the other 60% although they also have such cells in their bone marrow will be spared from relapse. Thus, demonstration of bone marrow epithelial cells is not sufficient for the individual patient to predict relapse [8]. It is possible that the route and time of seeding contribute to the final outcome. Therefore we have analyzed how surgery effects the level of circulating epithelial cells in different patients using our approach for real-time monitoring of changes in the number of circulating tumor cells [9,10]. These analyses may contribute to our understanding of how surgery influences the course of disease in breast cancer.

## Patients and methods

Fifty two consecutive patients with newly diagnosed breast cancer of all stages were included. Stages were as follows: 2 patients with DCIS, 24 patients with T1N0 and 2 patients with T1N1; 6 patients with T2N0 and 5 patients with T2N1; 2 patients with T3, 7 patients with T4, 2 relapsed patients, 2 patients after cryotherapy.

Once informed consent had been obtained from all participants, as required for ethics committee approval, peripheral blood anticoagulated with EDTA was drawn before each intervention such as surgery, at day 3(4) and day 7 after surgery and at intervals of several months thereafter. First, red blood cells were lysed using ammonium chloride, which was followed by a single centrifugation step. Then, the pellet of white cells was collected (in accordance with a previously described approach [10] and incubated with FITC-conjugated mouse anti-human epithelial antibody (HEA; Miltenyi Bergisch Gladbach, Germany) for 15 min in the dark and readjusted to 1 ml. 20 μl of this suspension of unfixed vital cells were used for measurements applied to a poly-L-lysine treated slide, which was analyzed using a Laser Scanning Cytometer^® ^(Compucyte Corporation, Cambridge, MA, USA) [10].

Measurements of a defined area were started when the cells had settled and took about 5 min according to cell density. For optimal measurements it was imperative to have a single cell suspension with about 2–3 cell diameters space between the cells. The adherent cells were measured using a Laser Scanning Cytometer (LSC^® ^Compucyte Corporation, Cambridge, MA, USA). The cells could easily and unequivocally be contoured using forward scatter as a thresholding parameter at 20× magnification. Background fluorescence was determined dynamically to calculate both peak and integral fluorescence on a per-cell basis. This unique method corrects for variation in background fluorescence and makes the fluorescent calculation equivalent for all cells. The FITC-HEA positive cell fluorescence was collected using a 530/30 nm bandpass filter and amplified using a photomultiplier (PMT). Values are displayed as scattergrams and histograms and percentages and mean values of positive and negative cells calculated from the region comprised of single cells only. The LSC^® ^enables the user to relocate cells contained within the positive population for visual examination through the microscope. In addition a CCD camera attached to the microscope allowed taking photo- and fluoromicrographs at the same time [9]. This method, termed MAINTRAC^® ^analysis, enables relocation of cells for visual examination discrimination of live and dying cells and quantification, and for taking fluoromicrographs. Statistics were calculated according to the student's T-test.

## Results

**Figure 1:**
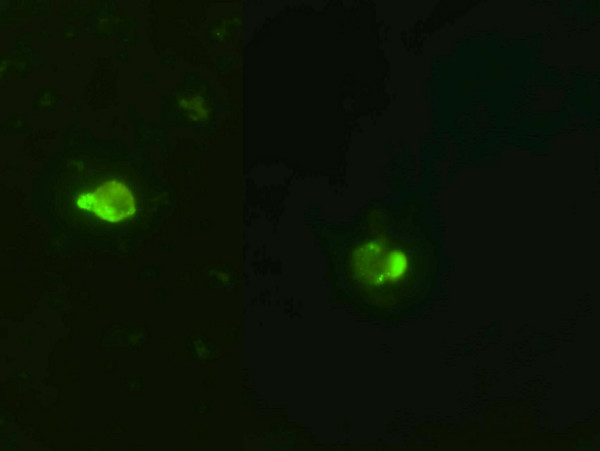
Positively stained gated green fluorescing cells which have been relocalized and visually analyzed. Typical pictures of such cells detected by their green fluorescing cap are shown.

Viability of the cells detected was always visually verified looking for exclusive surface staining but also substantiated by the long persistence in blood. Typical pictures of such cells detected by their green fluorescing cap are shown in Figure 1. Pre-therapy numbers varied considerably between patients from 47160 to 200 cells/ml. No correlation was found between tumor size or stage and number of circulating cells (table 1). It should be mentioned that two patients with DCIS, too, had considerable numbers of circulating epithelial cells already before surgery.

**Table 1:** Mean values of pre-surgery epithelial cell numbers of patients.

	**T1(n = 27)**	**T>1(n = 23)**	**p**
**Mean value**	6233 ± 5601	10818 ± 7102	0.1637
	**N0(69%)**	**N1-3(31%)**	**p**
**Mean value**	6756 ± 5624	12008 ± 8465	0.1672
	**ER+(81%)**	**ER-(19%)**	**p**
**Mean value**	9501 ± 7827	5955 ± 4935	0.4298
	**DCIS(n = 2)**		
**Mean value**	12100		

Mean values of pre-surgery epithelial cell numbers of patients with different tumor sizes, involvement of lymph nodes and expression of estrogen receptor

**Figure 2:**
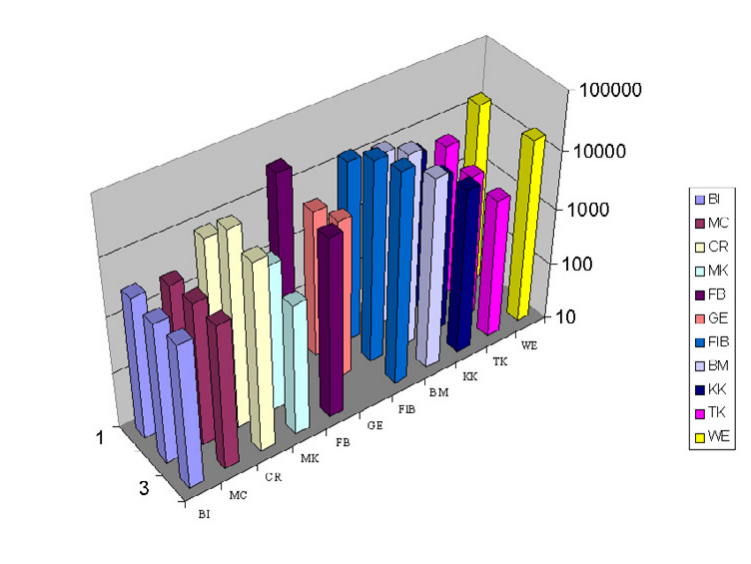
Analysis of the number of circulating cells from 11 patients immediately before and 30 and 60 min after surgery.

**Figure 3:**
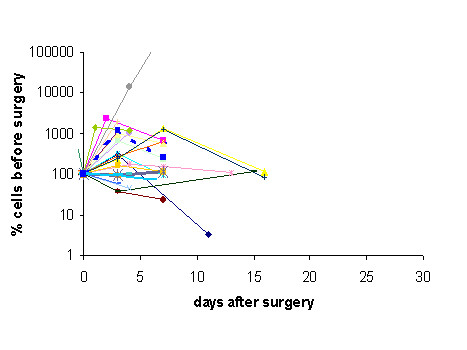
Increase and decrease of circulating cells of 25 patients before and 3 and 6 days after surgery for breast cancer setting pre-surgery values 100%. The fat dotted line with squares represents the mean increase and decrease of all 25 patients. Two patients (one during cryotherapy and one with DCIS (fat lines) retain identical numbers during intervention.

**Figure 4:**
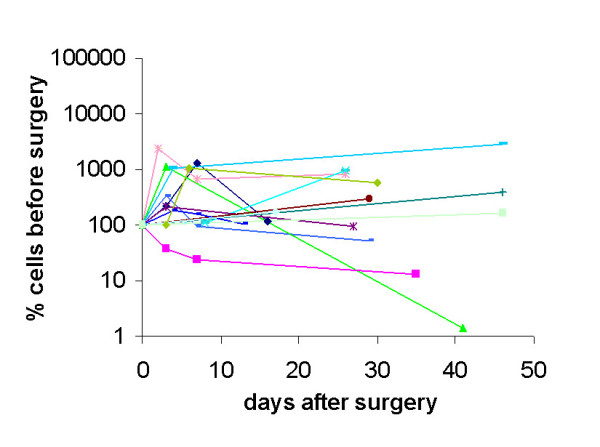
% of pre-surgery values of recirulating cells until onset of adjuvant chemotherapy.

Setting pre-surgery values 100%, cell numbers analyzed at every time point were normalized and the effect of surgery on circulating epithelial cell numbers was investigated. In 11 patients who were monitored before and 10 and 30 min after surgery the immediate release of cells during surgery was analyzed. As seen from Figure 2 there was almost no change in cell numbers during this early time after surgery. Surprisingly, however, cell numbers increased steeply in 85% of patients versus day 3 after surgery (Figure 3). Such an increase in circulating epithelial cells might be due to mobilization of normal epithelial cells during wound healing therefore cell numbers were again monitored 7 days after surgery. Indeed, there was a re-decrease in 58% of patients versus day 7 after surgery but pre-surgery values were reached only in 38% of cases (Figure 3). All other patients had cell numbers remaining above pre-surgery values. The two patients with DCIS and the two patients receiving cryotherapy belonged to the group of patients who did not increase in epithelial cell numbers after surgery but their numbers remained remarkably stable during this time (Figure 3 two fat lines). 15 patients could be further monitored until onset of adjuvant therapy. Only in four of them cell numbers decreased below pre-surgery values, in the others numbers remained stable after day 7 or even further increased (Figure 4). This indicates that the release of cells into circulation observed at day 3 is not only due to dying or normal adjacent cells rapidly eliminated from peripheral blood but obviously also long-lived cells are seeded into the circulation. These patients included 3 patients with advanced tumors (T2-4, N+) who already shortly after surgery started increasing their numbers in circulating epithelial cells and have relapsed in spite of adjuvant chemotherapy. Two patients with ER- tumors showed decreasing cell numbers and after adjuvant chemotherapy are still in complete remission.

**Figure 5:**
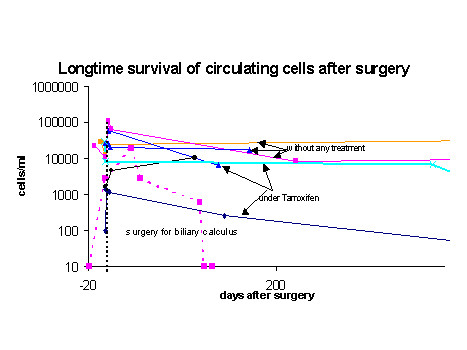
Numbers of longtime recirculating cells in patients without adjuvant chemotherapy. Two patients (orange and blue (DCIS) line) have now been monitored more than three years always revealing almost identical number of recirculating cells. In contrast, in a patient without a solid tumor (dotted line) who underwent surgery for a benign condition, cells returned to below the threshold of detection without further treatment.

Most patients received adjuvant therapy after surgery, therefore natural survival of circulating cells without treatment could be monitored only in 7 patients, in which no further treatment was given apart from Tamoxifen due to good prognostic markers. In these patients cell numbers remained high, decreasing slowly in 4 patients including one patient with DCIS but have been observed at the same high level now during three years in two patients without signs of relapse (Figure 5). This is in contrast to a patient without epithelial malignancy who has been operated on for biliary calculus. Before surgery no circulating epithelial cells were detected in her blood, increasing to high numbers right after surgery remaining high for more than three months, but then returned to below detectable numbers even in repeated analyses (Figure 5).

## Discussion

Circulating epithelial cells can be detected in many patients with solid tumors but rarely in healthy subjects [10]. Since most solid tumors are of epithelial origin or have an epithelial component, it is assumed that these circulating epithelial cells are shed from the tumor. Therefore, we investigated how different diagnostic and therapeutic interventions during the course of disease influence the number of epithelial cells in the blood stream. Circulating cells were present in almost all patients already before surgery.

At the analyses 10 and 30 min after surgery cell numbers remained at almost identical levels as before surgery indicating that there was no significant early wash out of cells into the circulation in the patients operated on in our institution as an immediate consequence of surgery. In contrast at day 3 to 4 after surgery, an increase in epithelial cell numbers was observed in the vast majority of patients. If normal surrounding cells were shed into circulation due to wound healing these cells would be expected to be cleared from the circulation rapidly. This was, indeed, the case for part of the cells until day 7. In some patients, however, cell numbers further increased and even in patients with re-decreasing cell numbers, pre-surgery values were rarely attained and levels of cell numbers remained elevated even after prolonged times independent of tumor size. Part of the cells seemed to remain stably in the circulation and this was true also for patients with DCIS. They may be malignant cells capable to survive. It is not known, to what extend they are able to form metastases [11] but if growing aggressively they might contribute to metastasis formation as seen in three patients with increasing cell numbers who suffered relapse. This may be one reason for the screening paradox of young breast cancer patients [12].

It has been claimed that tumor cells have a short half life [4]. If pre-surgery cell levels had been due to constant replenishing of the blood with cells from the tumor these cells would have been expected to rapidly decrease after removal of the primary tumor. Obviously, however, long-lived cells, presumably tumor cells, in addition to short-lived epithelial cells, were shed into the circulation probably already before surgery and following surgery and were detectable in the circulation even after extended times until adjuvant therapy.

Short lived epithelial cells would rather be expected to be normal cells shed into circulation during wound healing. This was substantiated by our observation during monitoring of a patient without an epithelial tumor. This patient had been monitored as a control with a systemic hematological disorder and been repeatedly negative, but suddenly showed a steep increase in circulating epithelial cells when she underwent surgery for a biliary calculus. Cell numbers returned to negativity only after about hundred days which is compatible with a more delayed wound healing after this treatment, but then remained negative.

Thus, even benign cells seem to be mobilized into circulation [13,14] and our results indicate that they are cleared from blood and cell numbers return to below detection after completion of wound healing. In breast cancer patients, in contrast, cell numbers remained elevated or even increased until adjuvant therapy. In the few patients with good prognostic markers who did not receive further therapy cell numbers remained at the same high level and in two patients have remained at this level even after three years of observation. Our results are in contradiction to the hypothesis, that tumor cells detectable even after 20 years of complete remission are constantly replenished from hidden micrometastases and rather favor the assumption that these cells can re-circulate in a "dormant" state for long times without leading to relapse, not only due to the inability of settled cells to form blood vessels [15] but also due to inefficiency of the preceding steps [16-18]. A single analysis detection circulating epithelial cells, in our opinion, therefore can not predict relapses. The present results, showing that patients with further increasing cell numbers after surgery, as previously also demonstrated for non small cell lung cancers [19], tend to relapse early, corroborate the requirement for repeated analysis.

We were not able to distinguish benign and malignant epithelial cells by surface antigen staining or morphology. Reports, observing a rapid decline of epithelial cells after surgery, therefore, might look at the elimination of benign and dying malignant cells. Gene analysis at that time may also include benign cells present around diagnosis and surgery and this may make results of genetic variability ambiguous [20].

The surgical management of breast cancer is rapidly evolving towards less invasive procedures [21]. Indeed, in two patients, treated with cryotherapy, no additional cells were released after this manipulation. The role of the released cells for the patient's course of disease is not clear but they might be the source of subsequent metastases [22]. According to our criteria of exclusive surface staining the cells were still viable. These cells are then the target of adjuvant therapy but such treatment might be inefficient as long as the cells are not in the cell cycle. Recent calculations indicate that these cells might grow in an irregular fashion [23] making repeated analyses necessary for monitoring their activity.

Tight tracing and quantification of circulating tumor cells might, therefore, it becomes an essential tool for therapy monitoring in solid tumors.

Moreover, peripheral blood being a transport medium with its load is influenced by influx from e. g. tumor and efflux into different organs, analysis of circulating tumor cells during tumor development may open a whole new field in experimental follow up of tumor progress and possibly metastasis development.

## Conflict of interest

Katharina Pachmann is holder of the patient for the method, termed MAINTRAC^® ^analysis. The authors declare that they have no competing interests

## Authors' contributions

**KP **performed the conception and designof the study and drafted the manuscript; **HN **designed the studies for the perioperative analyses; **OC **and **AK **carried out the study, and participated in its design and coordination; **MR **participated in the design of the study and performed the immunoassays; **CJ **performed the immunoassays. All authors read and approved final manuscript.

## References

[B1] Bonadonna G, Moliterni A, Zambetti M, Daidone MG, Pilotti S, Gianni L, Valagussa P (2005). 30 years' follow up of randomised studies of adjuvant CMF in operable breast cancer: cohort study. BMJ.

[B2] Schmidt-Kittler O, Ragg T, Daskalakis A, Granzow M, Ahr A, Blankenstein TJ, Kaufmann M, Diebold J, Arnholdt H, Muller P, Bischoff J, Harich D, Schlimok G, Riethmuller G, Eils R, Klein CA (2003). From latent disseminated cells to overt metastasis: Genetic analysis of systemic breast cancer progression. PNAS.

[B3] Pachmann K, Camara O, Kavallaris A, Schneider U, Schünemann S, Höffken K (2005). Quantification of the response of circulating epithelial cells to neodadjuvant treatment for breast cancer: a new tool for therapy monitoring. Breast Cancer Res.

[B4] Meng S, Tripathy D, Frenkel EP, Shete S, Naftalis EZ, Huth JF, Beitsch PD, Leitch M, Hoover S, Euhus D, Haley B, Morrison L, Fleming TP, Herlyn D, Terstappen LW, Fehm T, Tucker TF, Lane N, Wang J, Uhr JW (2004). Circulating tumor cells in patients with breast cancer dormancy. Clin Cancer Res.

[B5] Pachmann K (2005). Long-time recirculating tumor cells in breast cancer patients. Clin Cancer Res.

[B6] Demicheli R, Miceli R, Moliterni A, Zambetti M, Hrushesky WJ, Retsky MW, Valagussa P, Bonadonna G (2005). Breast cancer recurrence dynamics following adjuvant CMF is consistent with tumor dormancy and mastectomy-driven acceleration of the metastatic process. Ann Oncol.

[B7] Janni W, Rack B, Schindlbeck C, Strobl B, Rjosk D, Braun S, Sommer H, Pantel K, Gerber B, Friese K (2005). The persistence of isolated tumor cells in bone marrow from patients with breast carcinoma predicts an increased risk for recurrence. Cancer.

[B8] Braun S, Marth C (2004). Circulating tumor cells in metastatic breast cancer-toward individualized treatment?. N Engl J Med.

[B9] Pachmann K, Heiss P, Demel U, Tilz G (2001). Detection and quantification of small numbers of circulating tumour cells in peripheral blood using laser scanning cytometer (LSC). Clin Chem Lab Med.

[B10] Pachmann K, Clement JH, Schneider CP, Willen B, Camara O, Pachmann U, Höffken K (2005). Standardized quantification of circulating peripheral tumor cells from lung and breast cancer. Clin Chem Lab Med.

[B11] Naumov GN, Townson JL, MacDonald IC, Wilson SM, Bramwell VH, Groom AC, Chambers AF (2003). Ineffectiveness of doxorubicin treatment on solitary dormant mammary carcinoma cells or late-developing metastases. Breast Cancer Res Treat.

[B12] Baines CJ (2005). Are there downsides to mammography screening?. Breast J.

[B13] Cserni G, Bianchi S, Boecker W, Decker T, Lacerda M, Rank F, Wells CA, European Working Group for Breast Screening Pathology (2005). Improving the reproducibility of diagnosing micrometastases and isolated tumor cells. Cancer.

[B14] Diaz NM, Cox CE, Ebert M, Clark JD, Vrcel V, Stowell N, Sharma A, Jakub JW, Cantor A, Centeno BA, Dupont E, Muro-Cacho C, Nicosia S (2004). Benign Mechanical Transport of Breast Epithelial Cells to Sentinel Lymph Nodes. Am J Surg Pathol.

[B15] Naumov GN, Bender E, Zurakowski D, Kang SY, Sampson D, Flynn E, Watnick RS, Straume O, Akslen LA, Folkman J, Almog N (2006). A model of human tumor dormancy: an angiogenic switch from the nonangiogenic phenotype. J Natl Cancer Inst.

[B16] van Nimwegen MJ, Verkoeijen S, van Buren L, Burg D, van de Water B (2005). Requirement for focal adhesion kinase in the early phase of mammary adenocarcinoma lung metastasis formation. Cancer Res.

[B17] Aguirre-Ghiso JA, Ossowski L, Rosenbaum SK (2004). Green fluorescent protein tagging of extracellular signal-regulated kinase and p38 pathways reveals novel dynamics of pathway activation during primary and metastatic growth. Cancer Res.

[B18] Chambers AF, George N, Naumov GN, Vantyghem SA, Tuck AB (2000). Molecular biology of breast metastasis: Clinical implications of experimental studies on metastatic inefficiency. Breast Cancer Res.

[B19] Rolle A, Günzel R, Pachmann U, Willen B, Höffken K, Pachmann K (2005). Increase in number of circulating disseminated epithelial cells after surgery for non-small cell lung cancer monitore by MAINTRAC^® ^is a predictor for relapse: A preliminary report. World J Surg Oncol.

[B20] Schardt JA, Meyer M, Hartmann CH, Schubert F, Schmidt-Kittler O, Fuhrmann C, Polzer B, Petronio M, Eils R, Klein CA (2005). Genomic analysis of single cytokeratin-positive cells from bone marrow reveals early mutational events in breast cancer. Cancer Cell.

[B21] Singletary SE (2001). New approaches to surgery for breast cancer. Endocr Relat Cancer.

[B22] Engel J, Eckel R, Kerr J, Schmidt M, Furstenberger G, Richter R, Sauer H, Senn HJ, Holzel D (2003). The process of metastasisation for breast cancer. Eur J Cancer.

[B23] Retsky MW, Swartzendruber DE, Bame PD, Wardwell RH (1994). Computer model challenges breast cancer treatment strategy. Cancer Invest.

